# The effect of chitosan coating combined with cold plasma on the quality and safety of pistachio during storage

**DOI:** 10.1002/fsn3.3355

**Published:** 2023-04-06

**Authors:** Sahar Akhavan‐Mahdavi, Mehdi Mirzazadeh, Zahra Alam, Somaye Solaimanimehr

**Affiliations:** ^1^ Research Club, iQneiform Oy Juva Finland; ^2^ Department of Food Science and Technology, Faculty of Agriculture, Kermanshah Branch Islamic Azad University Kermanshah Iran; ^3^ Department of Chemistry, Faculty of Science Imam Khomeini International University Qazvin Iran; ^4^ Food and Drug Administration (FDA) Kermanshah University of Medical Sciences Kermanshah Iran

**Keywords:** aflatoxin, chitosan, cold plasma treatment, pistachio, storage

## Abstract

Pistachios are one of the most important agricultural and export products of Iran. Fresh pistachio fruit has soft skin, is highly perishable, and therefore has a short life after harvesting, which has made traders and consumers have a great desire to increase the shelf life of this product. For this purpose, in this study, the effect of different concentrations of chitosan as an edible coating (0.5 and 1.5% w/v) and the duration of cold plasma treatment (60 and 120 s) were investigated during 180 days of pistachio storage. The effect of treatments on the shelf life of pistachio fruit was evaluated by determining moisture content, color components, peroxide value, total mold and yeast, hardness, aflatoxin content, and sensory evaluations. The results showed that the treatment with 1.5% chitosan coating and 120 s of cold plasma treatment preserved the hardness of the pistachio and the color indices in the best way (*p* < .05). Also, this treatment had the minimum number of peroxide, aflatoxin, and mold and yeast counts during the storage time. The treatments with chitosan coating and under plasma application did not cause any unpleasant odor or taste during the storage time. In conclusion, according to the results of this research, it was determined that the simultaneous use of chitosan coating and cold plasma treatment can potentially be used as a new approach for commercial applications and the export of fresh pistachios.

## Graphical abstract

In this study, fresh pistachios were simultaneously exposed to cold plasma and chitosan coating. The results showed that plasma application for 120 s and 1.5% chitosan coating significantly reduced the amount of aflatoxin and peroxide number.

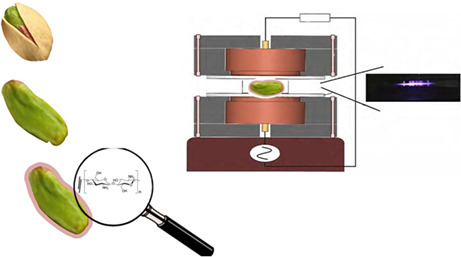

## 1 INTRODUCTION

The pistachio (*Pistacia vera*), a member of the cashew family, is one of the major export products of Iran. The high nutritional value and taste of pistachio nuts have led to the spread of their consumption in raw and roasted forms in different parts of the world (Razavi, 2005). Recently, due to the increase in pistachio cultivation areas in the world and the diversity of pistachios supplied to the world markets, there has been fierce competition between the exporting countries. Increasing health quality and low aflatoxin levels and the appearance of pistachios and proper packaging are important factors in this field (Cheraghali et al., 2007; Cheraghali & Yazdanpanah, 2010). During the storage period, mold and the production of toxins, especially aflatoxin, by insect attack, color change, absorption of foreign odors, texture destruction, and the appearance of old flavors and oxidation reactions cause a sharp and severe drop in product quality (Mohsen et al., 2020; Tavakolipour, 2015). Reducing the concentration of oxygen in the warehouse environment, changing the composition of gases in the warehouse space, introducing inert gases during packaging, and using edible coatings are among the most important methods that can be used to prevent these problems.

Edible coatings are thin layers of materials that create a barrier against the transfer of moisture, oxygen, and dissolved substances in food and prevent the absorption of moisture in dried fruits and products and the loss of moisture in fresh fruits and meat products (Suhag et al., 2020). These coatings can be edible and have different types, such as polysaccharides, proteins, lipids, and composites. If materials that have antioxidant and antimicrobial properties are used in the preparation of edible coatings, the shelf life of food materials will increase (Ju et al., 2019).

Chitosan, which is prepared from crab and shrimp waste, can be used as edible films. Due to the unique characteristics of chitosan and its beneficial nutritional effects, the US Food and Drug Administration has approved chitosan as a food additive (Mirbagheri et al., 2023). Several studies have used chitosan as an edible coating in nuts, including the use of chitosan in pistachios (Barzaman & Mirdehghan, 2018; Kaviani et al., 2015; Maghsoudlou et al., 2012; Mirbagheri et al., 2023; Rezaiyan Attar et al., 2022), walnuts (Sabaghi et al., 2015), almonds (Mirsharifi et al., 2022), cashew (Azimzadeh & Jahadi, 2018), and peanut (Kazemian‐Bazkiaee et al., 2020).

Decontamination of dry products such as pistachios is difficult because microorganisms, especially spore‐bearing microorganisms, are more resistant in an environment with low water activity. To sterilize and reduce the microbial load of pistachios, methods such as irradiation, and use of ethylene oxide, and steam are also used, and each of the mentioned methods has disadvantages. The radiation method is very expensive and people have a negative attitude toward it. Ethylene oxide is banned as a carcinogenic substance in the European Union. The steaming method also causes clumping, color change, etc. Considering the mentioned disadvantages and existing problems, there is a need to develop new processes to reduce the microbial load and replace the existing methods.

One of the developed antimicrobial technologies for sterilizing contaminated surfaces is the use of atmospheric nonthermal plasma. This technology, which is based on the use of ionized gases and produced at room temperature and atmospheric pressure, has been noticed since the mid‐1990s, but its use as a method of microbial decontamination of food still attracts the attention of researchers (Pankaj et al., 2018). Plasma is the fourth state of matter after solid, liquid, and gas. Plasma is created by the increase in molecular energy and the interaction of ionized particles, and these particles include atoms, free radicals, electrons, photons, and positive and negative ions. In order to produce plasma, ionized gases such as oxygen, air, nitrogen, or argon can be used under the influence of high‐frequency electromagnetic or electric fields and by placing in direct (or alternating) currents and waves (radio or microwave) (Bourke et al., 2018). The most common method to produce and stabilize a cold plasma is to apply an electric field to a neutral gas. This process may take place under atmosphere, vacuum, high pressure, heat, and chemical reactions. Cold plasma by electrons and ion bombardment, as well as the thermal effect and free radical production of ultraviolet rays, causes the destruction of the bacterial cell membrane and denatures the proteins, and also causes the destruction of DNA (Pankaj & Keener, 2017; Zhu et al., 2020). Cold plasma technology is considered one of the emerging alternative techniques for preserving food commodities, extending shelf life, and retaining bioactive compounds in foods. Besides, due to its nonthermal nature, CPT is a useful technology for the sterilization process, especially for heat‐sensitive foods (Siciliano et al., 2016; Wu et al., 2021). There are fewer reports on aflatoxins degradation by cold plasma. In particular, relevant research for aflatoxins degradation is still at the laboratory stage, with most experiments on vessels containing a few food samples (Wu et al., 2021).

Previous studies have used cold plasma to reduce the microbial load of different products (Guo et al., 2023; Mahnot et al., 2020; Mandal et al., 2018; Misra & Jo, 2017; Niveditha et al., 2021); however, no study has investigated the joint effect of coating with chitosan and cold plasma on pistachios. Therefore, as aflatoxins contamination is a vital challenge facing the pistachio as well as considering the importance of cold plasma technology and edible coatings as novel technologies to extend the shelf life of food products, the purpose of this study is to use the simultaneous application of plasma treatment and chitosan coating on pistachio and investigating the effect of these treatments on the physicochemical, microbial, sensory characteristics, and reducing aflatoxin of pistachio.

## 2 MATERIALS AND METHODS

### 2.1 Materials

Akbari variety pistachios were purchased from Rafsanjan, Iran. The hard shell of the pistachio was separated and the healthy kernels were selected and stored in polyethylene and black nylons in the refrigerator (4°C) until use. Chitosan powder with two low molecular weights (70 KD) from crab shells, each in 50‐g cans was purchased from Sigma Aldrich. The powders of aflatoxins were obtained from Sigma. All other chemicals used in this study were of analytical grade and purchased from chemical suppliers.

### 2.2 Preparation of chitosan solution

In order to prepare chitosan solutions with concentrations of 0.5% and 1.5% (w/v), respectively, 5 and 15 g of chitosan powder were added slowly and in several stages to 1000 mL of 1% acetic acid on a stirrer. Stirring continued for 5–6 h (until all chitosan particles were dissolved and the solution became clear). After the solution became clear, glycerol was added to the solution as a plasticizer equal to half the weight of chitosan and stirring was continued for another 15 min. Then the solution was removed from the magnetic stirrer and it reached the ambient temperature. After that, the solution was filtered with the help of Whatman no. 3 filter paper and a vacuum pump (Maghsoudlou et al., 2012).

### 2.3 Pistachio coating

At first, pistachios were weighed and placed in mesh containers. By placing the mesh container containing the pistachio kernels in the container containing the chitosan solution, the pistachio kernels were immersed in the chitosan solution for 40 s and removed. For this purpose, concentrations of 0.5%, 1%, and 1.5% of each type of chitosan with low molecular weights were used. The control sample was also prepared by immersing pistachio kernels in water. After coating, in order to remove excess moisture, the pistachios were dried for 4 h in an oven at 40°C (until the moisture content was 3.5% or 4%) (Zhang et al., 2017).

### 2.4 Cold plasma treatment

**FIGURE 1:**
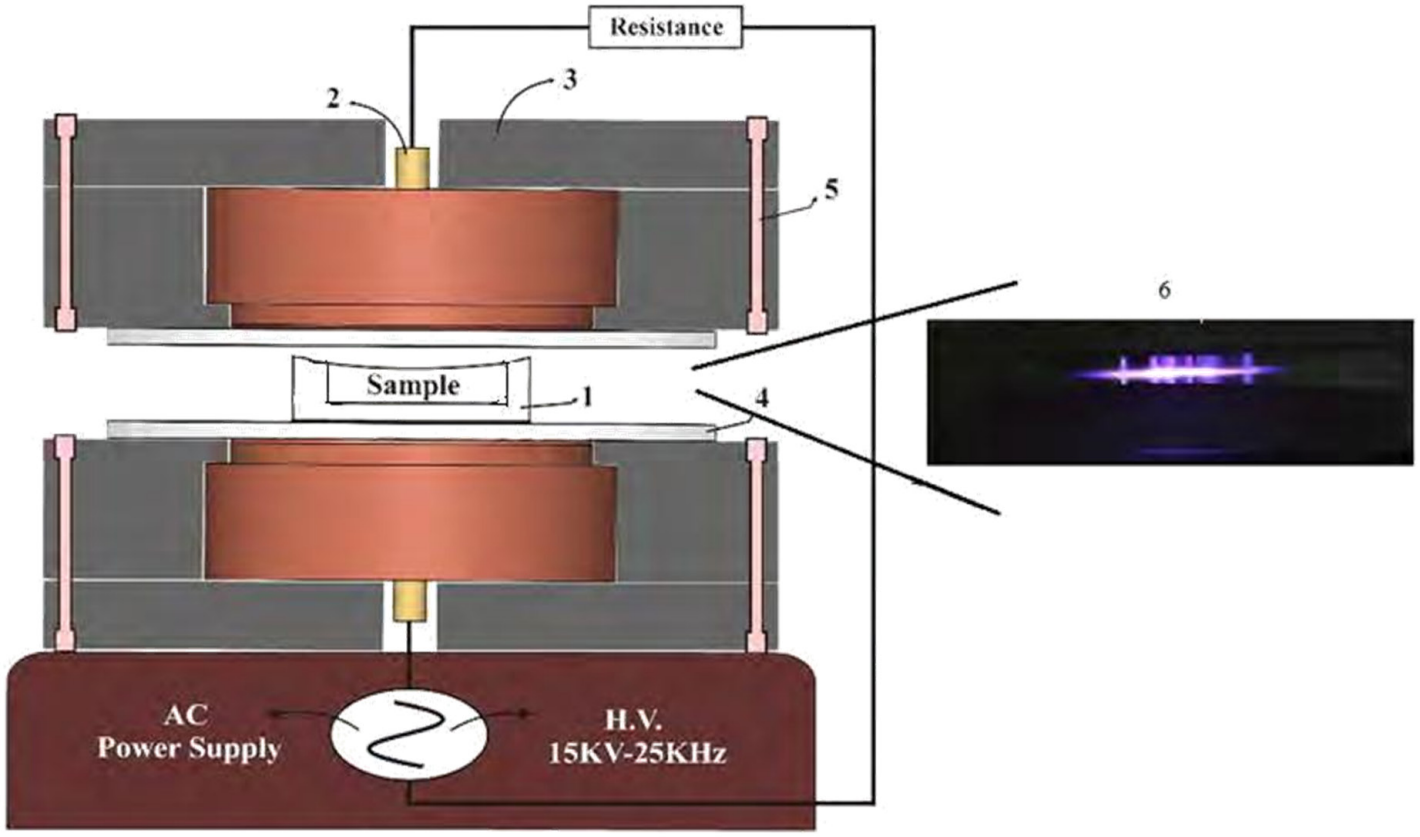
Schematic of the atmospheric dielectric barrier plasma production plan: (1) Location of the sample; (2) copper electrode; (3) plexiglass screen; (4) Glass screen; (5) screws; (6) Plasma flux on the sample.

The main structure of plasma products in this article is the discharge of the dielectric barrier (Nik Fanavaran Plasma, Iran). This structure includes two cylindrical copper electrodes with a height of 12 mm and a diameter of 40 mm. Each of the electrodes was mounted between two circular plates. Air was used as a gas source to form a uniform plasma in the space between two electrodes by applying a voltage between two electrodes. Argon gas type, oxygen gas pressure of 0.4 millibars equivalent to 0.3 Torr and power of 89 watts which were equivalent to radiometric waves. In order to treat pistachios with plasma, about 100 g of coated pistachios was placed in the machine, observing the hygiene requirements (Zhu et al., 2020). Then they were treated for 60 and 120 s (Figure 1).

### 2.5 Storage stability

The prepared pistachio kernels were randomly divided into 50 g units and packed in 20 × 6 cm polyethylene bags. The packed pistachio kernels were kept at room temperature (25–27°C) for 6 months. During the storage period, every month, one package was randomly taken from each treatment and chemical and microbial tests were performed on all treatments. The treatments used in this study are shown in Table 1.

**TABLE 1:** Treatments used in this study.

Treatments	Cold plasma treatment (P) S	Chitosan coating (C) %
P_0_C_0_	0	0
P_0_C_0.5_	0	0.5
P_0_C_1.5_	0	1.5
P_60_ C_0_	60	0
P_60_ C_0.5_	60	0.5
P_60_ C_1.5_	60	1.5
P_120_ C_0_	120	0
P_120_ C_0.5_	120	0.5
P_120_ C_1.5_	120	1.5

### 2.6 Moisture content

Moisture content was measured according to AOAC (2000) method. First, metal containers for measuring humidity were washed and placed in a 105°C oven for 2 h; then they were transferred to the desiccator and reached the ambient temperature. From each treatment, 5 g of pistachio nuts was crushed and after transferring to a container, their weight was recorded; then they were transferred to a 105°C oven. After 5 h, the dishes containing crushed pistachio kernels were removed from the oven and transferred to the desiccator; after reaching the ambient temperature, their weight was recorded. The moisture was obtained through Equation 1. The amount of moisture was reported based on wet weight.

(1) $$\text{Moisture}\;\left(\%\right)=\frac{M_{1}-M_{2}}{M_{0}}\times 100$$

where *M*_1_ is the weight of the dish and pistachio kernels before entering the oven at 105°C, *M*_2_ is the weight of the container and pistachio kernels after leaving the oven at 105°C and reaching the ambient temperature in the desiccator and *M*_0_ is the initial weight of pistachio nuts.

### 2.7 Color analysis

Color analysis measured by Hunter lab colorimeter (ColorFlex, USA).

### 2.8 Peroxide value (PV)

Before performing the peroxide value test, pistachio oil was extracted using Rabadán et al. (2017). The Peroxide value was determined according to the official methods of AOCS (2011). Briefly, the oil sample (3 g) was dissolved in glacial acetic acid (30 mL) and chloroform (20 mL) (3:2 v/v). Then saturated KI solution (1 mL) was added. The mixture was kept in the dark for 1 min, after adding distilled water (50 mL), the mixture was titrated against sodium thiosulfate (0.01 N). The PV value (mEq of oxygen/kg) was calculated using the following equation:

(2) $$\mathrm{PV}\;\text{value}=1000\;\left(S\times N\right)\times W$$

where S is the volume of sodium thiosulfate solution (blank corrected) in mL, N is the normality of sodium thiosulfate solution, and W is the weight of the oil sample (gram).

### 2.9 Total count of mold and yeast

For this purpose, 10 g of pistachio nuts was pounded with a sterile mortar next to the flame and under the microbial hood. Pistachio powder along with 90 mL of sterilized 0.85% sodium chloride solution was transferred to the Stomaker bag and mixed completely in the Stomaker. From dilutions 10^−1^, 10^−2^, and 10^−3^ were also prepared. Yeast Glucose Chloramphenicol Agar (YGC) culture medium was used for the cultivation and counting of mold and yeasts in pistachio samples. After preparing the culture medium, it was sterilized. After that, the temperature reached 45°C. Under the hood, 10 to 15 mL of the medium was added to the disposable plates, and a period of time was allowed for the medium to completely close in the plates. Using a sampler, the amount of 0.1 mL of each dilution was added to the solid culture medium and it was completely spread on the surface of the culture medium using a special glass rod. After a few minutes, the plates were kept upside down at room temperature and the plates were incubated at 20–25°C. After 5 days, the number of mold and yeast grown was counted (Maghsoudlou et al., 2012).

### 2.10 Hardness

The hardness of pistachios was measured with a TA‐XT2 texture analyzer (Stable Microsystems, Surrey, UK) provided by the software “Texture Expert”. An Aluminum 25 mm diameter cylindrical probe was used in a “Texture Profile Analysis” (TPA) double compression test to penetrate to 50% depth, at 1 mm/s speed test, with a 30 s delay between first and second compression (AACC method 74–09). In order to conduct the experiment, 15 samples of pistachio kernels without any type of defects from each treatment were tested by considering the instructions of the device. The texture test was done based on a single‐cycle compression test with a 20 mm diameter cylindrical probe and a probe speed of 50 mm per min (Khoshnoudi‐Nia & Sedaghat, 2019).

### 2.11 Determination of aflatoxins

The measurement of aflatoxin was done by HPLC method using immunoaffinity column clean up‐Test method (Balsini et al., 2021; Rezaie & Zareie, 2021). For minimizing the sub‐sampling error in aflatoxins analysis, water slurry of pistachio samples were prepared. For that matter, 1.5 L of water was added to 1 kg of pistachio. The resulting mixture was blended for 15 min with the slurry machine. Finally, 125 g of the test portion from the slurry was taken for the analysis. Each analysis was repeated three times and the average was reported. For purification and preconcentration of aflatoxins prior to the quantitative HPLC analysis, the immunoaffinity columns (Vicam Company, Water town, MA, USA) were used. Stock standard solution of aflatoxins with concentrations of 10 μg/mL was prepared in methanol. This standard was used to prepare mixed working standards for HPLC analysis. HPLC analysis was performed on a Waters (USA) HPLC system equipped with a Waters 600 pump, a Waters 600 column thermo‐controller, a Waters In‐line Degasser and Waters 474 Fluorescence Detector, and a Chromolith® performance RP18 analytical column (100 × 4.6 mm). The guard column was Chromolith® RP18. The fluorescence detector was operated at 365 and 435 nm for excitation and emission, respectively. UV‐visible spectra of aflatoxins stock solution were obtained using a Shimadzu UV‐1700 Pharma‐Spec spectrometer (Tokyo, Japan). Spectrophotometer equipped with a standard 10 mm path length spectrophotometer cell.

Pistachio slurries and 5 g NaCl were blended using a blender (Waring 8011S, Torrington, CT) with 400 mL of methanol: hexane (75:25) for 5 min to obtain a homogeneous sample mix. The mixture was centrifuged for 5 min at 448 *g*. The extract was filtered through filter paper (Whatman No. 4), then 20 mL aqueous methyl alcohol phase was mixed with 130 mL phosphate buffer saline (PBS) solution and filtered through a glass microfiber filter (Whatman, Inc. Clifton, NJ, USA) and 100 mL passed through an immunoaffinity column. Aflatoxins were eluted from the column by passing 1.5 mL of HPLC grade methanol and then 1.5 mL of HPLC grade water and using gravity to collect the eluate into a glass vial. A 50 μL aliquot of the eluate was injected into the HPLC.

### 2.12 Sensory analyses

Twenty trained panelists aged between 20 and 30 years were selected to take part in the sensory panel. The panel measured the selected critical pistachio attributes such as color, texture, flavor, appearance, and overall acceptance according to the five‐point hedonic scale (Moslehi et al., 2021).

### 2.13 Statistical analysis

Each experiment was carried out at least in duplicate and measurements were performed at least in triplicate. Statistical analysis of data was performed using Microsoft Excel. Analysis of variance was calculated using the SPSS (Version 26.0. Armonk, NY: IBM Corp.) with a confidence level of 0.05, to find any significant difference between treatments.

## 3 RESULTS AND DISCUSSION

### 3.1 Moisture

**FIGURE 2:**
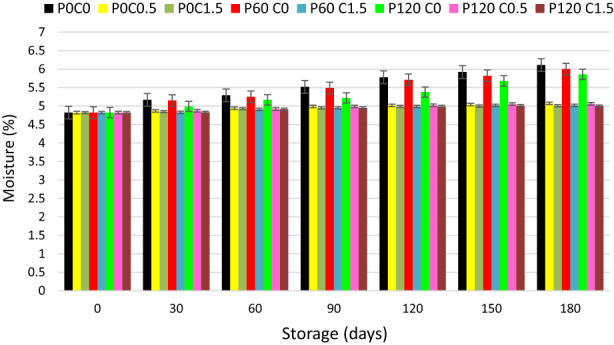
Moisture changes of pistachios treated with chitosan and plasma during storage.

The moisture content is one of the important factors in determining the quality of dried fruit. The moisture content of pistachios during harvesting is 35–40%, which decreases to 4–6% during the drying process; in this moisture, the product is stable and its water activity is less than 0.6. Changes in the moisture content of pistachio samples are shown in Figure 2. Due to the low initial moisture content of the product (4.82%) and the absence of a hard pistachio shell as a protector, there is a possibility of moisture absorption by the pistachio kernel during the storage period. By using edible chitosan coating and polyethylene bags as secondary packaging, the rate of moisture absorption in pistachio nuts can be reduced. During the storage period at room temperature and relative humidity (25–27°C and 35–45%), the amount of moisture absorption in the samples without chitosan coating was always higher than the samples with coating and a significant difference was observed between them, which is in agreement with the results of previous studies (Hamasalih & Rasul, 2022; Jafari & Javadi, 2020; Kaviani et al., 2015). Also, the moisture content of chitosan‐coated samples remained in the range of 4% (initial product moisture) from the beginning to the end of the storage period. While in the samples without chitosan coating, due to the lack of barrier against moisture transfer, at the end of the storage period, the pistachio kernel moisture content reached about 6%. As seen in Figure 2, changes in moisture with different plasma treatments do not follow a specific pattern. Therefore, it can be said that plasma treatment had no significant effect in preventing moisture transfer and only the presence or absence of chitosan coating was effective in moisture transfer. In general, the chitosan coating, like a barrier, reduces the rate of moisture transfer between pistachio kernels and the surrounding atmosphere. Due to the low moisture level of pistachio kernels and considering that the pistachio kernels were packed in polyethylene bags and were not in direct contact with the humidity of the environment, therefore chitosan concentrations did not show a significant effect on the moisture content of pistachios.

### 3.2 Color parameters

The color of the pistachio kernel is the natural color of the third skin of a healthy pistachio kernel, which usually varies depending on the variety, the length of growth, the time of picking, and storage, and is a mixture of green, purple pink, and red. Anthocyanin, chlorophyll, and xanthophyll are the main pigments of the pistachio kernel, and their amount varies depending on the ripening of the pistachio fruit (Molamohammadi et al., 2020). Since no adverse enzymatic or nonenzymatic reactions occur during the storage of dried pistachio nuts, the main reason for checking the color of pistachio nuts during storage was to ensure that the coating on the pistachio nuts was colorless and that the nuts did not change color by covering them with chitosan.

Table 2 shows the results of color parameters L*, a*, and b* of pistachios, which represent their lightness, redness, and yellowness, respectively, during 6 months of storage. As it is clear, with the passage of time, there was no clear increasing or decreasing trend in the value of a* and b* in any of the treatments while the amount of L* increased significantly. Therefore, the chitosan coating, which is a colorless and transparent liquid with no pigment, did not change color on pistachio kernels with the passage of time, so the color of the coated products in the last days of storage did not differ from the first days; which was consistent with the previous studies (Babapour et al., 2022; Sabaghi et al., 2015). The lighter color of pistachio kernels is probably due to the decomposition of mojo anthocyanins in pistachio kernels, including cyanidin glucoside and cyanidin galactoside over time. In the early stages of maintenance, no significant difference was observed between L* of different treatments. But gradually, from the third month onwards, the difference between them became significant. Also, according to the results of the plasma treatment, there was no effect on the color of any of the treatments.

**TABLE 2:** Color changes of pistachios treated with chitosan and plasma during storage.

Treatments	Storage (days)					
	0			180		
	Color parameters					
	L*	a*	b*	L*	a*	b*
P_0_C_0_	41.13 ± 0.62 ^a^	15.32 ± 0.84^a^	3.14 ± 0.36 ^a^	49.32 ± 0.84^a^	15.74 ± 0.52 ^a^	3.67 ± 0.06 ^a^
P_0_C_0.5_	41.13 ± 0.83 ^a^	14.25 ± 0.11 ^a^	2.96 ± 0.63^a^	48.25 ± 0.41 ^a^	14.55 ± 0.39 ^a^	4.00 ± 0.16^a^
P_0_C_1.5_	41.12 ± 0.62 ^a^	15.25 ± 0.96 ^a^	3.11 ± 0.55 ^a^	48.25 ± 0.16 ^a^	15.16 ± 0.49 ^a^	3.75 ± 0.31 ^a^
P_60_ C_0_	41.11 ± 0.27 ^a^	14.09 ± 0.04 ^a^	2.89 ± 0.37 ^a^	47.09 ± 0.73 ^a^	14.39 ± 1.12 ^a^	4.36 ± 0.81 ^a^
P_60_ C_0.5_	41.16 ± 0.67 ^a^	15.16 ± 1.04 ^a^	3.11 ± 0.73 ^a^	49.16 ± 1.16^a^	15.25 ± 0.49 ^a^	3.35 ± 0.38 ^a^
P_60_ C_1.5_	41.16 ± 0.36 ^a^	14.79 ± 0.35 ^a^	2.64 ± 0.23 ^a^	47.79 ± 0.35 ^a^	14.88 ± 0.39 ^a^	4.05 ± 0.18 ^a^
P_120_ C_0_	41.10 ± 0.16 ^a^	15.08 ± 0.62 ^a^	3.02 ± 0.35 ^a^	49.08 ± 0.22 ^a^	15.17 ± 0.05 ^a^	3.56 ± 0.26 ^a^
P_120_ C_0.5_	41.19 ± 0.24 ^a^	15.31 ± 0.14 ^a^	2.53 ± 0.16 ^a^	49.31 ± 0.13 ^a^	14.97 ± 0.44 ^a^	4.11 ± 0.73 ^a^
P_120_ C_1.5_	41.23 ± 0.43^a^	14.76 ± 0.17 ^a^	3.13 ± 0.36 ^a^	50.06 ± 0.17 ^a^	15.09 ± 0.64 ^a^	4.03 ± 0.36 ^a^

*Note*: Different letters indicate significant differences at the 5% level.

### 3.3 Peroxide value

**FIGURE 3:**
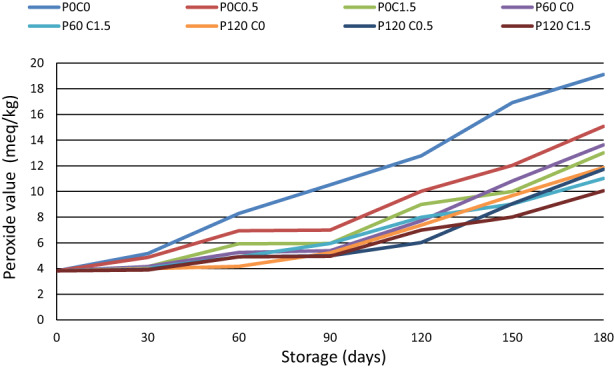
Peroxide value changes of pistachios treated with chitosan and plasma during storage.

Considering that about 53.5% of pistachio weight is made up of fat, and of this amount of fat, 56–70% is a monounsaturated fatty acid (oleic acid), and 18–31% is an unsaturated fatty acid (linoleic acid). This product is very ready for oxidation. If the environmental conditions are unfavorable during the pistachio storage period, spoilage reactions such as spontaneous, optical, and enzymatic oxidation begin and free radicals are formed. The production of these radicals, as well as the production of side compounds such as free fatty acids, hydroperoxides, aldehydes, ketones, and volatile alcohols, create an unpleasant taste and smell in the pistachio kernel (Kaviani et al., 2015). Peroxide value represents primary reaction products of lipid oxidation, which can be measured by their ability to liberate iodine from potassium iodide (Nor et al., 2008). According to Figure 3, during the storage period, the trend of changes in the peroxide value of all treatments was increasing; so in all the treatments, the peroxide number was the lowest in the first sampling and the highest in the last sampling. Another issue that should be noted is that in all treatments, pistachio kernels lacked a hard outer covering. The presence of this coating around the pistachio brain acts as a barrier against the penetration of moisture, oxygen, and metal ions into the brain tissue and reduces the speed of oxidative reactions. In all stages of storage, the highest amount of peroxide value was always related to the treatments without chitosan. These results indicate the effect of chitosan coating preventing the penetration of oxygen and moisture into pistachio brain tissue and its antioxidant property, which was consistent with the results of previous research (Hashemi et al., 2021; Jafari & Javadi, 2020; Kaviani et al., 2015). Chitosan is made from the de‐acetylation of chitin molecules. Chitosan chains containing glucosamine and ‐N‐acetylglucosamine molecules are connected to each other by hydrogen bonds. The presence of active sites and networks created by hydrogen bonds between chains causes the trapping of free radicals obtained from the initial stages of oxidation reactions and prevents the progress of these reactions. Also, chitosan, with its network structure and chelating properties, traps the catalysts of this reaction, especially metal ions. Metal ions decompose hydroperoxides and convert them into free radicals and accelerate radical chain reactions. By entrapping these catalysts in the chitosan network, the speed of oxidation reactions decreases. On the other hand, the chitosan coating on the pistachio kernel acts as a barrier, preventing the penetration of moisture, oxygen, and other catalysts into the pistachio tissue, and by protecting the pistachio kernel from various types of peroxides, it slows down the speed of oxidation reactions (Alsaggaf et al., 2017; Cai et al., 2014; Jonaidi Jafari et al., 2018). Another result obtained from this research was the relationship between chitosan concentration and its antioxidant effect. The samples coated with higher concentrations of chitosan had a lower peroxide number than the samples coated with a lower concentration of the same molecular weight. This problem indicates that with increasing chitosan concentration, its antioxidant effect increases.

Increasing the duration of plasma treatment has slightly increased the amount of peroxide index. The formation of lipid oxidation products in the plasma process increases with the increase in the application time and power of the plasma and the shelf life. The type of plasma source, the gas used, and the characteristics of the intended sample, including the amount of fat, play an important role in the amount of fat oxidation in nuts. According to Gavahian et al. (2018), the acceleration of the effect of cold plasma on lipid oxidation can be due to free radicals produced such as reactive oxygen and nitrogen species, excited atoms, ultraviolet rays, and charged particles in the plasma process. Reactive species, especially hydroxyl radicals, superoxide anion, and ozone, can initiate lipid oxidation reactions and, as a result, increase neck oxidation. In addition to the duration of plasma application, the structure of the sample also plays an important role in causing lipid oxidation (Gavahian et al., 2018).

### 3.4 Microbial count

**FIGURE 4:**
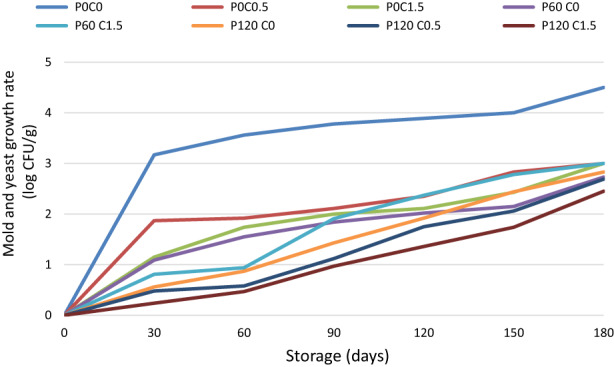
Total mold and yeast changes in pistachios treated with chitosan and plasma during storage.

During the storage period, the growth of mold and yeast in the sample showed an increasing trend. As seen in Figure 4, the sudden increase in the growth of mold and yeast started from the first month onwards. At the end of the storage period, the amount of mold and yeast reached 4.4 log cfu/g. This problem indicates that in the initial stages of storage of pistachio kernels, the microorganisms were in a delayed phase and no growth was observed in them, but gradually entered the logarithmic phase and their growth increased. The comparison of the average growth of mold and yeast of each treatment separately during the storage period showed that mold growth had the same speed throughout the storage period in samples containing edible chitosan coating. Also, there has always been a significant difference between the growth rate of mold and yeast of chitosan‐coated and uncoated samples. This shows that the chitosan coating has reduced the growth rate of the fungus in the pistachio kernel, which was consistent with the results of previous studies (Duran & Kahve, 2020; Molamohammadi et al., 2020; Moller et al., 2023). Lazaridou et al. (2007) stated that chitosan causes severe changes in the cell surface and covers the outer membranes with viscous structures. Therefore, chitosan, by binding to the outer membranes, causes the cell membrane to lose its function. This feature of chitosan is useful for food protection (Lazaridou et al., 2007). Mold cells are affected by chitosan's antimicrobial properties at any stage of growth; they stated that chitosan penetrates into the structure of microorganisms and prevents their cell division by binding to DNA molecules. In addition to the antimicrobial property of chitosan that was discussed, the presence of a layer of chitosan as an edible coating on each pistachio kernel acted as a barrier and prevented the growth of molds on the pistachio kernel in three ways: unfavorable conditions The growth of microorganisms by preventing the penetration of oxygen into the texture of the product, preventing the absorption of moisture by the product and increasing its water activity, physically preventing the penetration of microorganisms into the product (Goy et al., 2009; Hosseinnejad & Jafari, 2016). In addition, in the samples coated with higher concentrations of chitosan, less growth was seen in the amount of mold and yeast. This problem indicates that with increasing chitosan concentration, its microbial inhibitory effect increases. Considering that chitosan molecules have a positive charge due to free amino groups, with the increase in chitosan concentration, the positive charge on its molecules increases. This issue increases the rate of reactions between chitosan and bacterial and fungal cell walls (Hu & Gänzle, 2019).

The effect of the duration of plasma application and the duration of the storage period on the total count and the number of mold and yeast has been significant. Plasma application time is one of the important factors in the plasma process. Most of the studies conducted in this field are of the opinion that increasing the plasma radiation time increases the effect of the plasma. Zhuang et al. (2019), in a research, investigated the effect of plasma on the reduction of microbial load in chicken meat. They stated that by increasing the treatment time to 180 s, the microbial load decreased significantly (Zhuang et al., 2019). Cold plasma has the ability to produce particles and reactive species with antimicrobial properties such as hydroxyl radicals, hydrogen peroxide, oxygen molecules, superoxide anion, ozone, nitric oxide, nitrite, and ultraviolet waves. By attacking the cell wall, DNA, lipids, proteins and other cellular compounds, reactive oxygen, and nitrogen species cause damage to the microorganism cell and lipid oxidation, which causes disruption in the metabolic functions of the cell and as a result, the cell dies (Bagheri et al., 2020; Jerushalmi et al., 2020). In addition, the charged particles produced by the plasma are accumulated on the surface of the microbial cell and by creating a strong electric field, it tears the outer membrane and creates pores in the proteins. This leads to inhibition of enzyme activities and cell death. Ultraviolet rays have the ability to inhibit the growth of microorganisms due to the creation of thymine dimers in the nucleic acid of microorganisms. (Lee et al., 2016).

### 3.5 Hardness

**FIGURE 5:**
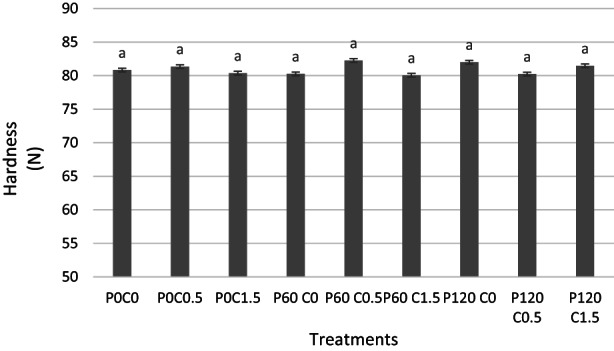
Hardness of pistachios treated with chitosan and plasma after 180 days of storage.

The results of pistachio hardness after 180 days of storage are shown in Figure 5. Accordingly, no significant difference was observed between the different treatments. In some studies, an increase in hardness in coated samples was reported, which is related to the higher moisture content (Li et al., 2022; Wang et al., 2015). Whereas some other studies did not observe a significant effect between chitosan coating and hardness (Kazemian‐Bazkiaee et al., 2020; Maghsoudlou et al., 2012).

### 3.6 Aflatoxin content

Aflatoxins are toxins that are mainly produced by a number of different species of Aspergillus such as *Flavus* and *Parasiticus* under certain conditions and have different types, the most important of which are B1, B2, G1, and G2. In this method, aflatoxins are meant to be B1, B2, G1, and G2 types. In the aflatoxin test, since it was not possible to analyze all the treatments, the ones that showed the best results in other tests, that is, P_120_ C_0_ and P_0_ C_1.5_ along with the control sample were analyzed on days 0, 90, and 120 (Iran, 2002; Regulation, 2010).

The results revealed (Table 3) that, the total aflatoxin levels in control treatment after 90 days of storage were above the Codex Committee on Food and Contaminants, the Iranian National Standard limit of 5 and 15 ng/g, and the European Union limit of 8 and 15 ng/g. Fathomer, the cold plasma treatment significantly had the lowest level of aflatoxin during storage.

**TABLE 3:** Color changes of pistachios treated with chitosan and plasma during storage.

Treatments	Total aflatoxin (ppb)		
	Storage (days)		
	0	90	180
P_0_C_0_	0.0341 ± 0.13^aA^	11.4951 ± 0.09^aB^	35.6811 ± 0.18^aC^
P_0_ C_1.5_	0 ± 0.0^aA^	3.4981 ± 0.19^bB^	4.7403 ± 0.06^bC^
P_120_C_0_	0 ± 0.0^aA^	1.0876 ± 0.27^cB^	1.1095 ± 0.15^cC^

*Note*: Different lowercase letters indicate significant differences between treatments (*p* > .001). Different capital letters indicate differences in meaning during storage (*p* > .001).

The antimicrobial effects of chitosan can be attributed to several internal and external factors, as well as a number of environmental factors, including pH, type of microorganism (fungal species), physiological state, molecular weight, concentration, degree of deacetylation, natural origin, physical form of chitosan and polycationic structure. Several studies have been conducted on the ability to remove aflatoxins by chitosan (Chaudhari et al., 2020; Cota‐Arriola et al., 2011).

In a similar study, Pirouz et al. (2020), investigated the removal of aflatoxins B1 and B2 in animal feed using chitosan. The results showed that the maximum reduction achieved with chitosan for aflatoxins B1 and B2 was 94.35% and 45.90%, respectively, under optimal conditions (Pirouz et al., 2020). Cota‐Arriola et al. (2011) investigated the antifungal effects of chitosan, as a natural substitute, on the growth of *Aspergillus parasiticus* and the production of aflatoxin B1. The results showed that the manufactured chitosan had fungicidal activity against *Aspergillus parasiticus* at the rate of 6.71 and 10.66 grams per liter and reduced the production of aflatoxin B1, which was consistent with the results of the present study (Cota‐Arriola et al., 2011).

Cold plasma as an emerging technique for the degradation of food contaminants attracted notable attention. According to the studies, cold plasma efficiently degraded many common pesticides and food allergens. These degradations occurred primarily due to the presence of reactive oxygen species (ROS) and reactive nitrogen species (RNS) in the plasma that attack the chemical bonds of food contaminants. The type of food contaminants that degrades are extremely dependent on the concentrations of plasma‐generated ROS and RNS.

Cold plasma treatment can decontaminate aflatoxins by blocking their production or degrading the final products, that is, cold plasma treatment can either inactivate aflatoxin and prevent aflatoxins contamination from the origin or convert aflatoxins into nontoxic or less‐toxic products. Research showed that several parameters, such as plasma generation device, plasma exposure time, plasma power, and the carrier gas composition, influence the type and concentration of reactive species (e.g., ROS and RNS) and the overall efficiency of cold plasma degradation for specific food contaminants (Wu et al., 2021). The aim of this study was to investigate the mechanism of degradation of food contaminants by cold plasma, and the benefits and drawbacks of this method (Tasouji et al., 2021).

Results available up to now show that the mechanisms of degrading aflatoxins by cold plasma treatment are mainly from the perspective of aflatoxins structure. Aflatoxins toxicity is related to the C8 = C9 double bond on the furan ring (toxicity site), and the degradation products are regarded as less toxic than the original due to losing the double bond (Tasouji et al., 2021; Wu et al., 2021).

### 3.7 Sensory evaluation

**FIGURE 6:**
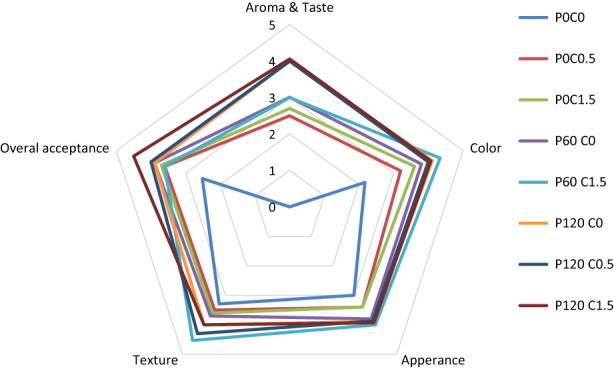
Sensory evaluation results of pistachio treated with chitosan and plasma after 180 days of storage.

The results of sensory analysis after 180 days of storage are presented in Figure 6 by a radar chart. The compared attributes were placed on pentagonal sides and the treatments were marked with different colors. The taste evaluation of the control sample was not done due to the contamination of the sample with aflatoxin, and this sample was rejected in terms of taste. According to the obtained results, it was found that in all the investigated parameters, the control sample and the plasma treatments received the lowest and highest scores, respectively. No significant difference was observed between different concentrations of chitosan and different times of plasma treatment. Chitosan is a tasteless and colorless compound, and in the concentrations used, the panelists did not feel any special taste or smell regarding the treatments containing chitosan. Sensory analyses of texture and color also confirm the results of instrumental analysis. In general, the evaluation of overall acceptance showed that the lowest and highest scores were related to the control sample and the P_120_C_1.5_ treatment, respectively.

## 4 CONCLUSION

The present study indicated that the cold plasma treatment and chitosan coating can be used as a potent method to increase the storage stability of pistachio. The results showed that the concentration of 1.5% chitosan and cold plasma treatment for 120 s could significantly reduce the amount of mold and yeast after 120 days of storage. It also had a significant effect on reducing the amount of aflatoxin. In addition, other physicochemical characteristics of pistachios did not change significantly. In addition, the sensory evaluation also showed that these treatments did not have an adverse effect on the sensory characteristics of pistachios. Thus, it successfully increased the shelf life of fresh pistachios. This study will provide bases for future studies in this area. It is recommended that other nuts and perishable food products be examined for microbial and aflatoxin content.

## AUTHOR CONTRIBUTIONS

**Sahar Akhavan‐Mahdavi:** Conceptualization (equal); data curation (equal); formal analysis (equal); funding acquisition (equal); investigation (equal); methodology (equal); project administration (equal); resources (equal); software (equal); supervision (equal); validation (equal); visualization (equal); writing – original draft (equal); writing – review and editing (equal). **Mehdi Mirzazadeh:** Data curation (equal); methodology (equal); software (equal). **Zahra Alam:** Investigation (equal); resources (equal); software (equal); writing – original draft (equal). **Somaye Solaimanimehr:** Formal analysis (equal); investigation (equal); software (equal).

## CONFLICT OF INTEREST STATEMENT

The authors have no conflicts of interest to declare. All co‐authors have seen and agree with the contents of the manuscript and there is no financial interest to report. We certify that the submission is original work and is not under review at any other publication.

## Data Availability

The data that support the findings of this study are available from the corresponding author upon reasonable request.
